# Factors Associated with Israeli Arab Women Anxiety and Depression During the COVID-19 Pandemic

**DOI:** 10.1007/s40615-024-01928-y

**Published:** 2024-04-10

**Authors:** O. Ali-Saleh, S. Bord, F. Basis

**Affiliations:** 1 Department of Health Systems Management, The Max Stern Yezreel Valley College, Jezreel Valley, 1930600 Israel; 2 Rambam Health Care Campus, 3109601 Haifa, Israel; 3 Technion Faculty of Medicine, Technion Israel Institute of Technology, 3200003 Haifa, Israel

**Keywords:** Anxiety, Depression, Arab minority, Women, Israel, COVID-19

## Abstract

**Background:**

Reports have shown that women suffered from anxiety, stress, depression, and fatigue during the COVID-19 pandemic more than men. No study so far has examined the effect of the pandemic among the Arab minority in Israel.

**Objectives:**

To examine the associations between levels of pandemic fatigue and stress of Israeli Arab women, and their anxiety and depression, along with their socio-demographic and socio-economic characteristics.

**Methods:**

A Cohen and Williamson questionnaire, which was based on a Likert scale, was distributed by the snowball method through social networks. Bivariate associations between the psycho-social and demographic characteristics and anxiety and depression were assessed using *t*-tests, chi-square tests, *Z* tests, and Pearson correlations. Multiple linear regressions were used to evaluate the associations with anxiety and depression, and the mediation model was examined with path analysis with bootstrapping.

**Results:**

Among 2294 Israeli Arab mothers who participated in the study, 63.7% were in the clinical range for anxiety, 67.4% for depression, and 57.5% for both anxiety and depression. Low economic status, pandemic fatigue, living in closed communities, and stress were related to anxiety and depression. Pandemic fatigue was positively related to stress, which was positively related to both anxiety and depression (standardized indirect effect = 0.137, *SE* = 0.014, 95%*CI* = 0.111, 0.164, *p* < .001; vs. 0.133, *SE* = 0.013, 95%*CI* = 0.108, 0.160, *p* < .001 respectively). The contribution of stress to anxiety and depression was significantly greater than that of pandemic fatigue (*Z* = 19.43 and *Z* = 18.04, *p* < .001, for anxiety and depression, respectively).

**Conclusions:**

Demographic characteristics may put Arab women at a higher risk of anxiety and depression. Elevated stress alongside high fatigue may trigger mental health difficulties. The welfare of minorities should be addressed by policymakers in relation to their demographic needs.

## Introduction

In March 2020, many countries took measures, such as lockdowns, wearing of face masks, and social distancing, to control the outbreak of the COVID-19 pandemic and protect their citizens. The pandemic, which lasted for over a year, increased the incidence of mental health conditions [1]. Around the world, pandemic fatigue was also reported to prevail as the pandemic lasted [2]. Pandemic fatigue is defined as a state of exhaustion and tiredness that may lead to distress and negatively affects emotions, cognition, and behavior. The official media in Israel dealt with the pandemic for at least 4 h a day for 2 years, not to mention the social media. Therefore, everyone, including women, suffered enormously from anxiety caused by the COVID-19 pandemic for a long period. Long-lasting stress may cause adaptation to and fatigue from the new situation, and hence indifference and less adherence to the guidelines [3]. More recent reports revealed higher levels of stress, anxiety, depression, and pandemic fatigue among women than men [4]. Compelling women to stay at home to take care of children and the elderly in the family resulted in workplace layoffs and reduced income for working mothers [5]. Furthermore, women had to deal with constant fear of staying at home (quarantine) with their infected children who were under the approved COVID-19 vaccination age (at the time of the study), as those children were somehow the source of outbreaks [6].

Many studies have dealt with anxiety and depression among women in general during the pandemic, but only a few of these studies have dealt with these mental states among women in specific minorities, while accounting for their demographic characteristics. In their study, Özdin S. et al. reported that women living in urban areas in Turkey and with a previous history of psychiatric illness were at risk of anxiety and depression [7]. According to this study, urban areas are more crowded, with residents living close to each other and more prone to infection than in rural areas, where people live in private houses in less crowded villages. In Poland, as the severity of loneliness increased, anxiety and depression among women increased [8]. A study of Jordanian citizens (Arab country) revealed that among those who were afraid of contracting COVID-19, there were higher levels of distress among men than women, but the levels of anxiety and depression were higher among women than among men [9]. Reports from Egypt (Arab country) showed a high rate of violence against Arab women during the COVID-19 pandemic, leading to women having to cope with fear and being deemed less functional in running their families [10].

The Arab ethnic minority in Israel is about 21% of the entire population, and most of the Arabs live in rural areas, such as villages and closed communities in the northern part of Israel [11]. In closed communities, sons, daughters, and their offspring live in close distance and sometimes in the same block with their parents. The relations between citizens are very warm and social. Therefore, they share knowledge and feelings, and they are more connected via social media, with less privacy. For the Jewish ethnic group, who are the majority population in Israel, most of the children live far from their parents and social relations are confined to smaller groups.

About 50% of Israeli Arabs have lower-than-average socio-economic status [11]. During the COVID-19 pandemic, Israeli Arabs had a high incidence of confirmed COVID-19 cases because of large social activities within the community, such as large weddings and funerals, and lower rates of vaccination [12, 13]. They also suffered from insufficient healthcare services with psychological and physical barriers, as they live in small villages on the periphery of the country [13]. Arabs in general have strong connections with their extended families, even if some live in urban areas. As mentioned above, these connections are more prominent in villages, where most Arabs live close to their parents and grandparents. Hence, during the pandemic, Arab women were overwhelmed by taking care of the sick and/or quarantining children/elderly family members, and they might have found it more challenging to adjust to the evolving restrictions, with high stress due to multiple responsibilities [14].

While some sought to improve the health situation by complying with instructions from the Israeli Ministry of Health (MOH), others were driven by care for their entire families and coping with rural social norms. A survey of the general population in Israel, with an emphasis on unique populations, revealed that the rates of the four kinds of violence (verbal, physical, financial, and sexual) within families increased significantly among the Arab minority compared to the Jewish majority. This may be related to socio-economic gaps and demographic characteristics [15].

Although the gap between Arabs and Jews childbirth is decreasing in the last decade, the birth rate is still higher among Arabs, especially among Bedouin Arabs, where it is quite twice that of the Jews [16]. Due to the closure of schools by the Israeli Ministry of Education, children stayed home, which amounted to more responsibility for their mothers. Therefore, mothers had to make sure their children attended classes via online platforms such as Zoom software [17]. This meant that mothers had to dedicate more time helping their children with schoolwork and setting up the technology for that. The responsibilities were even more challenging among the Arab minority, with a higher average number of children than in the general population, slow internet speed in most Arab villages, and the low socio-economic situation making it harder for children to have access to computers at home [18].

This study was undertaken after Israel offered free COVID-19 vaccination that reached over 50% of the general adult population above 18 years old [19]. By March 2021, after a year of living through three waves of COVID-19 (in Israel), there was a need to assess peoples’ feelings and mental health status during such a long and stressful pandemic in order to develop coping strategies to address their needs [20]. Since the Arab society in Israel have different demographic characteristics, understanding factors related to anxiety and depression among Arab women is important for developing specific plans to meet their specific needs. So far, no study has examined the needs of women in the Arab minority in Israel. Thus, development of programs tailored to this segment of the population by the Israeli MOH with the aid of local authorities/municipalities, such as deployment of public health professionals who speak the Arabic language, including community nurses and social workers, to deal with stress, boost resilience, and reduce anxiety and depression levels, has been somehow intuitive.

The aim of this study was to measure the prevalence of pandemic fatigue, stress, anxiety, and depression among Arab women, as their demographic conditions are different from the rest of the population, and to find out the main obstacles they faced due to these mental states in order to suggest ways to overcome them. Furthermore, other countries with large minorities may draw from this study to address similar issues in future pandemics.

The main objective of this study is to examine the associations between levels of pandemic fatigue and stress of Israeli Arab women, and their anxiety and depression, along with their socio-demographic and socio-economic characteristics, and more specifically, (1) to assess the socio- demographic and socio-economic associations with the clinical classifications of anxiety and depression; (2) to evaluate the extent to which pandemic fatigue and stress are associated with anxiety and depression, beyond the socio-demographic and socio-economic characteristics; and (3) to estimate the extent to which the women’s stress levels mediate the association between pandemic fatigue on the one hand, and anxiety and depression, on the other.

### Objectives

To examine the associations between levels of pandemic fatigue and stress of Israeli Arab women, and their anxiety and depression, along with their socio-demographic and socio-economic characteristics.

## Methods

An online quantitative questionnaire was compiled based on a reliable questionnaire used in previous studies [21]. The questionnaire was composed of several known and valid questionnaires assessing the variables of the study. To ensure reliability of the questionnaire, all items from the English version were translated into Arabic and back into English by Arabic language experts. The questionnaire was anonymous and participants were pre-informed that they had the right not to answer the questionnaire or to withdraw from the study at any time. The questionnaire was distributed using a snowball method, via social media platforms like Instagram, Facebook, and WhatsApp. The initial participants were asked to share the link to the questionnaire with other Israeli Arab women.

To test the dependent variable, i.e., the level of depression and anxiety, the PHQ-4 questionnaire was used. The PHQ-4 questionnaire contains four items and was constructed from a larger questionnaire, the PHQ-9 [22]. It examines depression (the GAD-7 questionnaire examines anxiety) [23]. The first two items measure depressed mood and loss of interest (anhedonia), of which at least one item is required to diagnose major depression disorder (MDD) according to the DSM-IV. The following is a sample item: “I have little interest or pleasure in doing things.” The PHQ-2 score of each question ranges from 0 to 6, and a cross-sectional point ≥ 3 indicates significant clinical depression. The last two items measure anxiety. The following is a sample item: “I am not able to stop, or control, worrying.” A cut-off score of 3 or above indicates anxiety. The PHQ-4 questionnaire does not diagnose clinical depression; however, it is a tool through which depressive tendencies or anxiety can be detected.

The mediator variable, level of stress (PSS-10—Perceived Stress Scale), was evaluated using the Cohen and Williamson questionnaire (1988) [21], which examines the extent to which an individual estimates situations and feelings in their life as stressful while accounting for the general stress level in recent months (in our case the last months of the pandemic). The questionnaire includes 10 items. The scores for each item range from 0 to 4, where higher scores indicate higher levels of stress. A total score of 0–13 is considered a low stress level, 14–26 a moderate stress level, and 27–40 a high stress level.

The questionnaire used to evaluate pandemic fatigue (an independent variable) comprised eight statements that were compiled based on the recommendations of the World Health Organization [24]. Some sample items include the following: “I’m tired of following the guidelines,” “The State / Ministry of Health did not explain the logic behind the guidelines,” “There are conflicting messages about the guidelines from various government bodies.” Respondents were asked to rate their levels of agreement with these statements on a Likert scale, where 1 indicates strongly disagree and 5 indicates strongly agree [25]. A CFA calculated for this questionnaire, using AMOS software ver. 29, showed a good fit (NFI = 0.974, TLI = 0.964, CFI = 0.977, RMSEA = 0.055), attesting to the construct validity of the questionnaire. A convergent validity was shown by Brazier et al. in a previous study [26].

Participants were finally asked to provide the following details about themselves: age, sex, religion (Muslim, Bedouin, Druze, or Christian), degree of religiosity (secular, traditional, religious, or very religious), marital status, number of children, place of residence (north, south, or central Israel), level of education (high school, post-secondary vocational education, or academic), type of employment (self-employed, employee, or unemployed), and income level (lower than average, average, higher than average)—according to a scale of the Central Bureau of Statistics [27]. The questionnaire was distributed in March–April 2021, after obtaining the approval of the Ethics Committee of the Max Stern Yezreel Valley College (YVC-EMEK 2021–52).

Data were analyzed with SPSS version 27. Internal consistencies were calculated for the study variables with Cronbach’s *α* (or Spearman’s correlations for two items), and the variables were composed with summations or averages of items. Demographic and background variables were described with means and standard deviations, or frequencies and percentages, and compared by the clinical cut-off points for anxiety and depression using *t*-tests, chi-square tests, and *Z* tests for the significance of the difference between independent proportions.

Means, standard deviations, and Pearson’s correlations were calculated for the study variables. Two multiple linear regression models were built to assess the extent to which the background variables and the study variables were related to anxiety and depression. The background variables were entered in step 1, and the study variables in step 2. Mediation was examined with path analysis, using AMOS version 27. Chi-square, NFI, NNFI, CFI, and RMSEA were used as measures of fit. Education level and economic status were controlled for. These control variables were allowed to correlate with each other, and so were the two dependent variables. Mediation was examined with bootstrapping of 2000 samples and bias-corrected 95% confidence interval. Continuous variables were standardized. The Bonferroni criterion for multiple comparisons was applied per table.

Cronbach’s *α* for the dependent variables was as follows: for anxiety *r* = 0.79 (*p* < 0.001) (two items, total range 0–6, clinical cut-off ≥ 3) and for depression *r* = 0.72 (*p* < 0.001) (two items, total range 0–6, clinical cut-off ≥ 3). In addition, Cronbach’s *α* for stress (an independent/mediating variable) was high (*α* = 0.85); the total score ranged between 0 and 40, with cut-off points of 0–13 indicating low stress, 14–26 indicating moderate stress, and 27–40 indicating high stress. Cronbach’s *α* for pandemic fatigue (eight items) was *α* = 0.85. All variables were defined such that higher scores represented greater values of the phenomena.

## Results

Two thousand, two hundred and ninety-four (2294) Israeli Arab mothers with young children participated in this study. Their age ranged from 20 to 45 years old, with a mean age of 32.24 years (*SD* = 6.30), and each mother had up to nine children (*M* = 2.57, *SD* = 1.32). Most were Moslem (92.3%) and married (96.7%). About 52% of the participants had an academic education, 9% were undergraduate students, 19% had more than a high school education but not an academic education, and 20% had up to 12 years of education. About 55% of the participants lived in rural areas, mostly in the northern part of Israel (60%). About 57% of them were religious, and 38% were partly religious. The participants reported low (48%), average (39%), or good (13%) economic status. About 13% of them reported that they had been sick with COVID-19 (Table 1).

**Table 1:** Background characteristics by the clinical cut-off points for anxiety and depression (*N* = 2294)

	Socio-demographic and background characteristics							
	Total sample		Anxiety, non clinical (*n* = 832)	Anxiety, clinical (*n* = 1462)		Depression, non clinical (*n* = 748)	Depression, clinical (*n* = 1546)	
Mean age (*SD*), range	32.24 (6.30), 20–45		32.63 (6.37)	32.02 (6.24)	*t*(2292) = 2.26 *p* = .024	32.51 (6.32)	32.11 (6.28)	*t*(2292) = 1.43 *p* = .152
Religion (%)								
Moslem	2117 (92.3)		759 (35.9)	1358 (64.1)	*χ*^2^(2) = 2.95 *p* = .228	676 (31.9)	1441 (68.1)	*χ*^2^(2) = 7.73 *p* = .021
Christian	109 (4.8)		42 (38.5)	67 (61.5)		40 (36.7)	69 (63.3)	
Druze	68 (2.9)		31 (45.6)	37 (54.4)		32 (47.1)	36 (52.9)	
Marital status (%)								
Married	2218 (96.7)		806 (36.3)	1412 (63.7)	*χ*^2^(2) = 0.38 *p* = .827	724 (32.6)	1494 (67.4)	*χ*^2^(2) = 0.17 *p* = .918
Divorced	62 (2.7)		22 (35.5)	40 (64.5)		19 (30.6)	43 (69.4)	
Widow	14 (0.6)		4 (28.6)	10 (71.4)		5 (35.7)	9 (64.3)	
Mean number of children (*SD*), range	2.57 (1.32), 1–9		2.57 (1.29)	2.57 (1.33)	*t*(2292) = 0.05 *p* = .958	2.55 (1.28)	2.58 (1.34)	*t*(2292) = − 0.62 *p* = .532
Education (%)								
Up to 8 years	33 (1.4)		7 (21.2)	26 (78.8)	*Z* = 11.38 *p* < .001 (academic vs. non-academic)	5 (15.2)	28 (84.8)	*Z* = 19.37 *p* < .001 (academic vs. non-academic)
8 to 12 years	432 (18.8)		141 (32.6)	291 (67.4)		126 (29.2)	306 (70.8)	
Higher education	428 (18.7)		138 (32.2)	290 (67.8)		112 (26.2)	316 (73.8)	
BA student	218 (9.5)		75 (34.4)	143 (65.6)		72 (33.0)	146 (67.0)	
BA	807 (35.2)		293 (36.3)	514 (63.7)		282 (34.9)	525 (65.1)	
MA and above	376 (16.4)		178 (47.3)	198 (52.7)		151 (40.2)	225 (59.8)	
Area of residence (%)								
Northern Israel		1380 (60.2)	527 (38.2)	853 (61.8)	*χ*^2^(2) = 5.52 *p* = .063	463 (33.6)	917 (66.4)	*χ*^2^(2) = 1.81 *p* = .405
Central Israel		755 (32.9)	252 (33.4)	503 (66.6)		232 (30.7)	523 (69.3)	
South Israel		159 (6.9)	53 (33.3)	106 (66.7)		53 (33.3)	106 (66.7)	
Type of residence (%)								
Urban		1037 (45.2)	373 (36.0)	664 (64.0)	*Z* = 0.27 *p* = .786	322 (31.1)	715 (68.9)	*Z* = 1.44 *p* = .149
Rural		1257 (54.8)	459 (36.5)	798 (63.5)		426 (33.9)	831 (66.1)	
Religiosity (%)								
Secular		132 (5.8)	56 (42.4)	76 (57.6)	*χ*^2^(3) = 8.95 *p* = .030	48 (36.4)	84 (63.6)	*χ*^2^(3) = 7.66 *p* = .054
Partly religious		863 (37.6)	291 (33.7)	572 (66.3)		252 (29.2)	611 (70.8)	
Religious		1214 (52.9)	445 (36.7)	769 (63.3)		417 (34.3)	797 (65.7)	
Orthodox		85 (3.7)	40 (47.1)	45 (52.9)		31 (36.5)	54 (63.5)	
Economic status (%)								
Below average		1100 (48.0)	349 (31.7)	751 (68.3)	*χ*^2^(2) = 22.09 *p* < .001	330 (30.0)	770 (70.0)	*χ*^2^(2) = 10.24 *p* = .006
About average		890 (38.8)	347 (39.0)	543 (61.0)		298 (33.5)	592 (66.5)	
Above average		304 (13.2)	136 (44.7)	168 (55.3)		120 (39.5)	184 (60.5)	
Sick with COVID-19 (%)								
No		2004 (87.4)	714 (35.6)	1290 (64.4)	*Z* = 1.67 *p* = .094	642 (32.0)	1362 (68.0)	*Z* = 1.53 *p* = .125
Yes		290 (12.6)	118 (40.7)	172 (59.3)		106 (36.6)	184 (63.4)	

High percentages were found for the clinical ranges of anxiety and depression. About two-thirds of the mothers (*n* = 1462, 63.7%) were classified in the clinical range for anxiety. About two-thirds of the mothers (*n* = 1546, 67.4%) were classified in the clinical range for depression. About 57.5% (*n* = 1318) of the mothers were positive for both anxiety and depression, 16.2% (*n* = 372) were positive for either mild anxiety or mild depression, and 26.3% (*n* = 604) were classified below the clinical range for both anxiety and depression. About 21.6% of the mothers (*n* = 496) were classified as experiencing a high level of stress, 61.6% (*n* = 1414) as experiencing a moderate level of stress, and 16.7% (*n* = 384) as experiencing a low level of stress.

Applying the Bonferroni criterion for multiple comparisons (*p* < 0.005; Table 1), two significant socio-demographic differences were found based on the clinical cut-off points for anxiety and depression. Women with an academic education were less likely to be classified in the clinical range for anxiety and depression than women with an education level lower than academic education. Furthermore, women of above-average economic status were less likely to be classified in the clinical range for anxiety and depression than women of below-average economic status (marginal significance for depression).

Distributions of means, standard deviations, and correlations for the study variables revealed moderate means for the study variables (anxiety, depression, stress, and pandemic fatigue). High positive correlations were found between anxiety, depression, and stress, with low-moderate positive correlations between them and pandemic fatigue (Table 2).

**Table 2:** Means, standard deviations, and correlations for the study variables (*N* = 2294)

	*M* (*SD*)	2	3	4
1. Anxiety (0–6)	3.32 (1.93)	.82*	.65*	.24*
2. Depression (0–6)	3.42 (1.85)		.64*	.26*
3. Stress (0–40)	21.01 (7.78)			.22*
4. Pandemic fatigue (1–5)	3.38 (0.92)			

*p < .001

As mentioned above (Table 1), higher education level and economic status were less related to anxiety and depression. Education level was used as a dichotomous variable (0, below academic education; 1, academic education) while economic status (five categories), which did not deviate from a normal distribution (skewness = 0.21, *SE* = 0.05), was used as a continuous variable. Both were significantly related to the continuous variables anxiety (*r* =  − 0.08 and *r* =  − 0.13, *p* < 0.001) and depression (*r* =  − 0.09 and *r* =  − 0.12, *p* < 0.001). In light of these significant relationships, education level (0, below academic education; 1, academic education) and economic status (1, very bad; 5, very good) were used as control variables in further analyses.

Two multiple linear regression models were built to assess the extent to which the background variables, pandemic fatigue, and stress were related to anxiety and depression (Table 3). The resulting models were found significant and explained about 44% and 43% of the variance in anxiety and depression respectively. Applying the Bonferroni criterion for multiple comparisons (*p* < 0.01), we found that of the background characteristics, economic status was significant, as lower economic status was related to higher levels of anxiety and depression. Furthermore, higher levels of pandemic fatigue and stress were related to higher levels of anxiety and depression. The regression coefficients for economic status and pandemic fatigue were low, while the coefficient for stress was high. Thus, the contribution of stress to anxiety and depression was significantly greater than that of pandemic fatigue (*Z* = 19.43 and *Z* = 18.04, *p* < 0.001, for anxiety and depression, respectively).

**Table 3:** Multiple regressions for anxiety and depression with pandemic fatigue and stress (*N* = 2249)

	Anxiety			Depression		
	*β*	*p*	ΔAdj. *R*^2^	*β*	*p*	ΔAdj. *R*^2^
Step 1			.02			.02
Education level (academic)	− .05	.016	(< .001)	− .06	.003	(< .001)
Economic status (high)	− .12	< .001		− .11	< .001	
Step 2			.42			.41
Education level (academic)	− .01	.435	(< .001)	− .02	.138	(< .001)
Economic status (high)	− .07	< .001		− .06	< .001	
Pandemic fatigue	**.10**	** < .001**		**.12**	** < .001**	
Stress	**.63**	** < .001**		**.61**	** < .001**	
Total adj. *R*^2^			.44			.43
*F*(4, 2289)	456.70		(< .001)	432.57		(< .001)

**Fig. 1:**
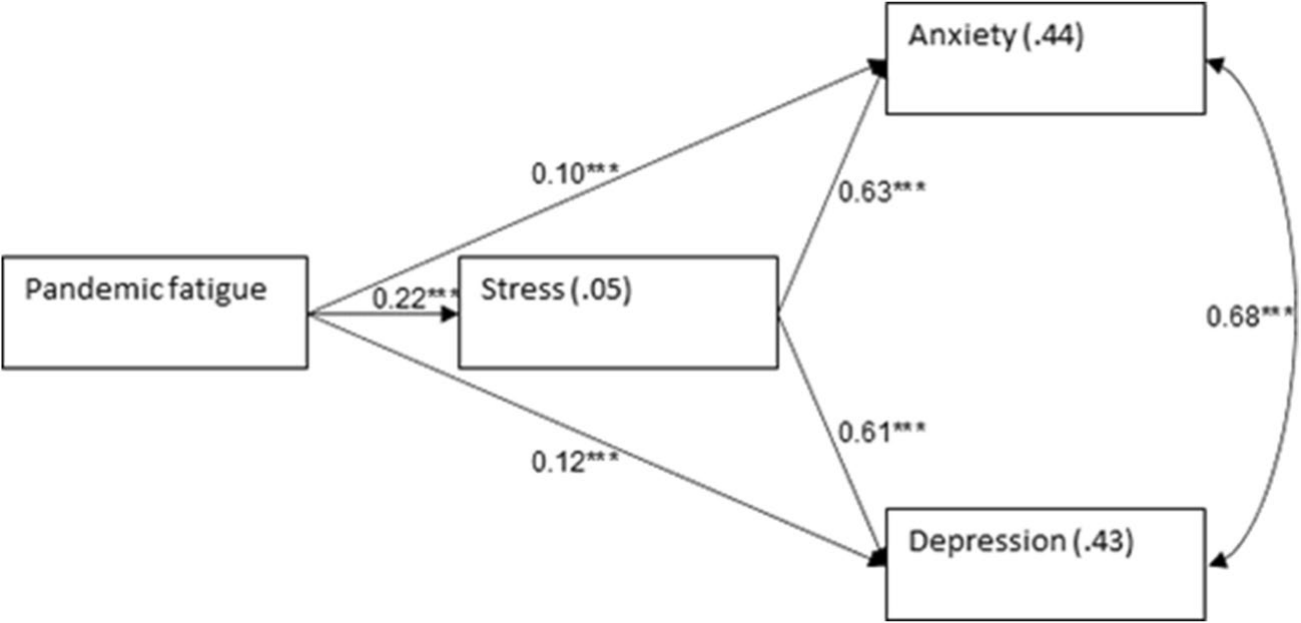
Path analysis for anxiety and depression with stress and pandemic fatigue. Note. *R*.^2^, values within rectangles; *β*, standardized regression coefficients—values related with one-way arrows; *r*, correlations alongside two-way arrows. ***p < .001

To assess the extent to which the level of stress mediated the relationship between pandemic fatigue and both anxiety and depression, a path model was examined with path analysis using AMOS version 27. Education level and economic status were controlled for; pandemic fatigue was defined as the independent variable and stress as the mediator. The control variables were allowed to correlate with each other, and so were the dependent variables. Continuous variables were standardized. Mediation was examined with bootstrapping of 2000 samples and bias-corrected 95% confidence interval. For the sake of clarity, the control variables are not shown in Fig. 1. The model was found to fit the data: *χ*^2^(3) = 5.36, *p* = 0.147, NFI = 0.999, NNFI = 0.997, CFI = 0.999, RMSEA = 0.019. Figure 1 shows the results of the path analysis.

Results showed that pandemic fatigue was positively related to stress, which in turn was positively related to both anxiety and depression. The mediated relationship between pandemic fatigue and each of anxiety and depression was found significant in the path model (for anxiety: standardized indirect effect = 0.137, *SE* = 0.014, 95%*CI* = 0.111, 0.164, *p* < 0.001; for depression: standardized indirect effect = 0.133, *SE* = 0.013, 95%*CI* = 0.108, 0.160, *p* < 0.001). In line with the mediation, the coefficients for the direct effects were low. Thus, higher pandemic fatigue was related to a higher level of stress, which in turn was related to higher levels of anxiety and depression.

## Discussion

Two thousand, two hundred and ninety-four (2294) Israeli Arab mothers with young children participated in this study. Their age ranged from 20 to 45 years old, with a mean age of 32.24 years (*SD* = 6.30), and each mother had up to nine children (*M* = 2.57, *SD* = 1.32). Most were Moslem (92.3%) (82.9% among the entire Arab population). The overall birth rate among Arab women in Israel is 2.98 live births per woman (as of 2019), compared to 2.51 among the Jewish population. Furthermore, there are significant differences among Arabs in different geographical areas. For example, the birth rate for women among the Bedouin community (5.26) is almost twice as high as that for Arab women in northern Israel [28].

Studies conducted in Israel showed that the compliance with the authorities’ instructions and restrictions was lower among the Arab minority in Israel than among the entire population [29]. Some studies have measured the prevalence of stress, anxiety, and depression among the entire population in Israel. These studies used data from central psycho-social services in the four HMOs that serve all Israeli citizens. They dealt with the number and diagnosis of citizens who were referred for these services but not the reasons for the referrals. Psycho-social services are underdeveloped in Arab rural areas, and Arabs are less likely to seek help because of fear of stigma from fellow Arabs in the same communities. This point led us to make an anonymous questionnaire, in Arabic language, to find out the prevalence of anxiety, stress, and depression among this population and the main reasons for this prevalence. Since there were no similar studies for the entire population, we could not compare our results to that of the entire population.

Post-traumatic stress disorder, depressive disorder, and anxiety disorder, as well as grief-related symptoms, have different effects on different population groups in various ways [30]. In 2020, the Israeli Central Bureau of Statistics (CBS) conducted a survey regarding aspects of residents’ resilience to the effects of the pandemic among all populations in Israel. The rate of women who reported feelings of isolation, stress, anxiety, and fear of COVID-19 was higher than the rate of men. Data analysis by Israel’s Health Maintenance Organizations (HMOs) revealed that 68% of telephone callers from the entire population who requested for mental support were women [30]. Women represented 60% of new patients in mental health clinics in 2019 and 2020. As the COVID-19 pandemic dragged on, more patients requested for mental health support [30]. This data was in line with the findings of the European Union (EU) that less women felt optimistic about the future than men [31].

In our study, about 83% of women suffered high and moderate levels of stress. About two-thirds suffered from anxiety, and about two-thirds suffered from depression. Reports to the Knesset (Israeli parliament) revealed that 68% of all referrals to mental clinics in the entire Israeli population (men and women) suffered from depression [32]. Although we do not have the whole data from this report, it seems that depression among Arab women was much higher than that among the general population (60% among men and women).

In our study, women with academic education were less likely to be classified in the clinical range for anxiety and depression than women with a lower education level. About 77% of the Arab population are educated up to matriculation level, and only 15% hold an academic degree. By contrast, 33% of the Jewish population have an academic degree [16]. These gaps in education have implications not only for Arab citizens’ prospects of entering the workforce but also for their potential earning power and working conditions. Unemployment and low salaries (economic resilience) may explain some of our findings.

A study by the Bank of Israel emphasizes how the poor representation of Arabs in high-tech jobs is due to gaps in education settings. The difficulties are not only encountered when trying to get into the industry, but also in the quality of employment and wages. High-tech jobs in the security fields in Israel are very limited and unavailable for the Arab minority in Israel because they do not serve in the Israeli Army [33]. On the other hand, working in the health sector is considered among the Arab population as prestigious and, therefore, the percentage of Arabs employed in this field is high. Government statistics from 2019 show that 50% of Arab women work either in education (31%) or in healthcare (19%) compared to 37% among the Jewish population (19% in education and 18% in healthcare) [34]. These professions might have put them in an increased risk of contracting COVID-19 (school pupils and sick patients). This, partially, may also explain the higher rate of anxiety among Arab women in this survey, a finding corroborated by Labrague’s study involving nurses in Indonesia [35].

Data from Israel’s National Insurance Institute show that most women’s employment was terminated, or they were put on leave of absence, during the pandemic compared to men [36]. Due to the closure of educational institutions during the pandemic, the long stay of children at home increased the tedium of household chores such as cooking and cleaning [37]. Parameters like lower income, spending more time in unpaid work, having more children than the general population, living in rural areas with elderly family members, and working more in education and medical professions where they are prone to get infected might have rendered Israeli Arab women more susceptible to anxiety and depression.

During the COVID-19 pandemic, a sample test by the Israeli Ministry of Social Services and Welfare found a 25–30% increase in referrals to centers for the prevention of domestic violence. Moreover, the police experienced an increase in the number of reports dealing with domestic violence mainly against women [15]. While police data revealed a relatively small increase in the number of complaints by Arab women (4%), data from the Ministry of Social Services and Welfare on inquiries to domestic violence centers by Arab families showed a much higher increase [15]. The gap between these data, each of which reflects a different aspect of the issue, raises a question regarding the accessibility of all treatment channels for Arab women (physical boundaries) and some cultural issues. In this regard, cultural issues refer to the fact that Arabs in Israel live in closed communities in villages, and contacting the police is perhaps often discouraged for fear of shaming and hurting the family’s reputation. This conflict between the needs of Arab women victims and the social needs of their extended families could further increase anxiety among these women. Although we did not examine this sensitive issue in our study, the authors assume that this issue must have influenced the mental health of women in Arab communities, and should be addressed by social authorities.

We also examined the relation between pandemic fatigue, stress, anxiety, and depression. As stated above, pandemic fatigue is a state of exhaustion and tiredness that may lead to distress and negatively affects emotions, cognition, and behavior [38]. It also may lead to a state of indifference, adaptation to a new situation, higher risk of depression, hopelessness, and lower compliance with the official guidelines. Our study revealed a positive correlation between pandemic fatigue and high levels of stress, which might have led to anxiety and depression (Fig. 1). In a previous study, Ali-Saleh et al. showed a positive correlation between the use of social media and pandemic fatigue. Both the use of social media, which was a mediating factor for pandemic fatigue, and pandemic fatigue itself were accompanied by lower trust in institutions among the Arab minority in Israel [13]. This might have led also to low compliance of Arab women with vaccinating their children [39].

In view of this, besides addressing the educational and socio-demographic needs of Israeli Arab minorities, it is important to consider sex factors through psycho-social interventions, in order to address physical boundaries, ease access to these interventions in Arabic language, and prevent pandemic fatigue among women, thus improving their welfare.

## Conclusion

The Arab minority in Israel has specific needs different from that of the entire population because of their unique demographic and psycho-social characteristics, and their low educational and economic status. This is more prominent among women and mothers. These women do unpaid household jobs, take care of more children than the average mother, incurred high expenses (during the pandemic) in order to purchase new computers for their children’s remote learning, battled poor internet connectivity, and take care of elderly extended family members, such as their own and their husbands’ grandparents.

The dilemma of the educational and economic status is an issue that should have been addressed in advance by the authorities.

Many obstacles should have been addressed in this pandemic. We believe that these obstacles may apply to other minorities in other countries as well.

### Limitations of the Study

This study focuses on anxiety among Arab women in Israel, but it does not compare this segment of the population with the general population because of lack of data. For future studies, we suggest comparing the differences in the levels of anxiety, depression, and pandemic fatigue between Arab women (the minority) and Jewish women (the majority) in Israel, since both groups of women have different socio-economic status, educational backgrounds, and demographic characteristics.

Another limitation of the study is that it examines the effect of pandemic fatigue on the development of anxiety and depression but not vice versa. For future studies, we suggest examining the effect of stress and anxiety on the development of pandemic fatigue, as persistent stress may lead to pandemic fatigue. If this is the case, then reducing stressing factors in pandemics, for instance by moderating declarations by the authorities and official media as well as by finding ways to reduce the spread of false news on social media, may reduce pandemic fatigue and increase compliance with guidelines given by the authorities [40].

## Data Availability

All data generated or analyzed during this study are included in this published article,

## References

[1] Panchal N, Kamal R, Cox C, Garfield R 2021. The implications of COVID-19 for mental health and substance use. Kaiser Family Foundation. 2021. https://www.kff.org/coronavirus-covid-19/issue-brief/the-implications-of-covid-19-for-mental-health-and-substance-use/.Accessed 9 Nov 2021.

[2] World Health Organization. Timeline: WHO’s COVID-19 response. 2020. https://www.who.int/emergencies/diseases/novel-coronavirus-2019/interactive-timeline.

[3] Smout A. Anxiety surged during pandemic, particularly among women – study. REUTERS. 2021. https://www.reuters.com/business/healthcare-pharmaceuticals/anxiety-surged-during-pandemic-particularly-among-women-study-2021-10-08/

[4] Pedrosa AL, Bitencourt L, Fróes ACF, Cazumbá MLB, Campos RGB, de Brito SBCS, Silva Simões E, AC. Emotional, behavioral, and psychological impact of the COVID-19 pandemic. Front Psychol. 2020;11:566212. 10.3389/fpsyg.2020.566212. 33117234 10.3389/fpsyg.2020.566212 PMC7561666

[5] Zalis S. Moms are less likely to return to the workforce post-COVID—here’s how employers can help. FORBES. 2021. https://www.forbes.com/sites/shelleyzalis/2021/09/09/moms-are-less-likely-to-return-to-the-workforce-post-covid-heres-how-employers-can-help/?sh=4de20f0c4d72. Accessed Mar 2023.

[6] Parshley L. Exclusive: Kids catch and spread coronavirus half as much as adults, Iceland study confirms. National Geographic. 2020. https://www.nationalgeographic.com/science/article/we-now-know-how-much-children-spread-coronavirus. Accessed Nov 2022.

[7] Özdin S, Bayrak Özdin Ş. Levels and predictors of anxiety, depression and health anxiety during COVID-19 pandemic in Turkish society: the importance of gender. Int J Soc Psychiatry. 2020;66(5):504–11. 10.1177/0020764020927051. 32380879 10.1177/0020764020927051 PMC7405629

[8] Idzik A, Leńczuk-Gruba A, Kobos E, Pietrzak M, Dziedzic B. Loneliness and depression among women in Poland during the COVID-19 pandemic. Int J Environ Res Public Health. 2021;18(20):10698. 10.3390/ijerph182010698. 34682443 10.3390/ijerph182010698 PMC8535819

[9] Abuhammad S, Khabour OF, Alomari MA, Alzoubi KH. Depression, stress, anxiety among Jordanian people during COVID-19 pandemic: a survey-based study. Inform Med Unlocked. 2022;30:100936. 10.1016/j.imu.2022.100936. 35399332 10.1016/j.imu.2022.100936 PMC8977212

[10] El-Nimr NA, Mamdouh HM, Ramadan A, El Saeh HM, Shata ZN. Intimate partner violence among Arab women before and during the COVID-19 lockdown. J Egypt Public Health Assoc. 2021;96(1):15. 10.1186/s42506-021-00077-y. 34132902 10.1186/s42506-021-00077-y PMC8206903

[11] Chernichovsky D, Bisharat B, Bowers L, Brill A & Sharony C The health of the Arab Israeli population. Taub Center for Social Policy Studies In Israel. Updated 2022 Apr 29. https://www.taubcenter.org.il/wp-content/uploads/2020/12/healthofthearabisraelipopulation.pdf.

[12] Efrati I Israeli Arab death rate from COVID-19 was three times higher than general population, study finds. HAARETZ. 2021. https://www.haaretz.com/israel-news/study-israeli-arab-death-rate-from-covid-three-times-higher-than-general-population-1.9937934

[13] Ali-Saleh O, Bord S, Basis F. Factors associated with decisions of Arab minority parents in Israel to vaccinate their children against COVID-19. Vaccines (Basel). 2022;10(6):870. 10.3390/vaccines10060870. 35746479 10.3390/vaccines10060870 PMC9227855

[14] Lavie E, Elran M, Sawaed K, Abu Mokh M, Dallashi M: Israel’s Arab society and the coronavirus challenge. INSS Insight No. 1288, March 31, 2020. https://www.inss.org.il/publication/coronavirus-and-the-israeli-arabs/. Accessed Jan 2023

[15] Resnikovski-Kuras A, Bachar Y, Arazi T. Domestic violence during the COVID-19 crisis stage II: unique groups and follow-up analyses. https://brookdale.jdc.org.il/en/publication/domestic-violence-during-covid19-stage-ii/. Accessed May 2023

[16] Haddad Haj-Yahya N, Khalaily M, Rudnitzky A, Fargeon B. Statistical report on Arab society in Israel. https://en.idi.org.il/articles/38540 . Accessed May 2023

[17] Paltiel O, Hochner H, Chinitz D, Clarfield AM, Gileles-Hillel A, Lahad A, et al. Academic activism on behalf of children during the COVID-19 pandemic in Israel; beyond public health advocacy. Isr J Health Policy Res. 2021;10:48. 10.1186/s13584-021-00485-7 10.1186/s13584-021-00485-7 PMC8371603 34407864

[18] World Bank Group. Lessons from remote learning during COVID-19. 2021. https://www.worldbank.org/en/topic/edutech/brief/how-countries-are-using-edtech-to-support-remote-learning-during-the-covid-19-pandemic. Accessed Jan 2023.

[19] Israel Ministry of Health (MOH) (2021). The coronavirus dashboard. https://datadashboard.health.gov.il/COVID-19/general. Accessed Nov 2022.

[20] Ekka-Zohar A, Kertes J, Cohen-Lunger E, Novikov I, Shamir-Stein N, Hermoni-Alon S, Mizrahi-Reuveni M. COVID-19 seropositive rates between the waves Israel. Isr Med Assoc J. 2021;23(10):611–4. 34672439

[21] Williamson G. Perceived stress in a probability sample of the United States. In: Spacapan S, Oskamp S, editors. The social psychology of health. Sage Publishers; 1988.

[22] Löwe B, Wahl I, Rose M, Spitzer C, Glaesmer H, Wingenfeld K, Schneider A, Brähler E. A 4-item measure of depression and anxiety: validation and standardization of the Patient Health Questionnaire-4 (PHQ-4) in the general population. J Affect Disord. 2010;122(1–2):86–95. 10.1016/j.jad.2009.06.019. 10.1016/j.jad.2009.06.019 19616305

[23] Delgadillo J, Payne S, Gilbody S, Godfrey C, Gore S, Jessop D, Dale V. Brief case finding tools for anxiety disorders: validation of GAD-7 and GAD-2 in addictions treatment. Drug Alcohol Depend. 2012;125(1–2):37–42. 10.1016/j.drugalcdep.2012.03.011. 22480667 10.1016/j.drugalcdep.2012.03.011

[24] World Health Organization. Pandemic fatigue Reinvigorating the public to prevent COVID-19. 2020. https://apps.who.int/iris/bitstream/handle/10665/335820/WHO-EURO-2020-1160-40906-55390-eng.pdf.

[25] Jebb AT, Ng V, Tay L. A review of key Likert scale development advances: 1995–2019. Front Psychol. 2021;4(12):637547. 10.3389/fpsyg.2021.637547. 10.3389/fpsyg.2021.637547 PMC8129175 34017283

[26] Brazier JE, Harper R, Jones NM, O’Cathain A, Thomas KJ, Usherwood T, Westlake L. Validating the SF-36 health survey questionnaire: new outcome measure for primary care. BMJ. 1992;305(6846):160–4. 10.1136/bmj.305.6846.160. 1285753 10.1136/bmj.305.6846.160 PMC1883187

[27] Central Bureau of Statistics. https://www.gov.il/en/Departments/news/press_06072022_c. Accessed Nov 2022.

[28] Statistical Report on Arab Society in Israel. 2021. https://en.idi.org.il/articles/38540#:~:text=The%20overall%20fertility%20rate%20among,differences%20among%20different%20geographic%20areas.

[29] Ali-Saleh O, Obeid S. Compliance with COVID-19 preventive guidelines among minority communities: the case of Israeli Arabs. J Racial Ethn Health Disparities. 2022;9:1–12. 10.1007/s40615-022-01344-0. 35679011 10.1007/s40615-022-01344-0 PMC9179224

[30] Guessoum SB, Lachal J, Radjack R, Carretier E, Minassian S, Benoit L, Moro MR. Adolescent psychiatric disorders during the COVID-19 pandemic and lockdown. Psychiatry Res. 2020;291:113264. 10.1016/j.psychres.2020.113264. 32622172 10.1016/j.psychres.2020.113264 PMC7323662

[31] European Parliamentary Research Service, The coronavirus crisis: an emerging gender divide?. 2022. https://www.europarl.europa.eu/RegData/etudes/ATAG/2021/679100/EPRS_ATA(2021)679100_EN.pdf.

[32] Avner I and Schwartz R: Arab society in the shadow of the corona pandemic, a report to the Israeli Knesset. 2022. https://fs.knesset.gov.il/globaldocs/MMM/ec77910d-2dac-eb11-8111-00155d0aee38/2_ec77910d-2dac-eb11-8111-00155d0aee38_11_17972.pdf.

[33] Nathaniel Gams: Only 1% of Arabs work in high -tech - and they also suffer from tens of percent wage gaps. The Marker. 2022 https://www.themarker.com/technation/2021-10-05/ty-article/.premium/0000017f-dbb1-d856-a37f-fff17af40000.

[34] Jerry Almo-Capital: Data on Arab women’s employment with emphasis on Bedouin on the Negev. 2022. https://fs.knesset.gov.il/globaldocs/MMM/9eea2a62-9152-ec11-813c-00155d0824dc/2_9eea2a62-9152-ec11-813c-00155d0824dc_11_19443.pdf .

[35] Labrague LJ. Pandemic fatigue and clinical nurses’ mental health, sleep quality and job contentment during the covid-19 pandemic: the mediating role of resilience. J Nurs Manag. 2021;29(7):1992–2001. 10.1111/jonm.13383. 34018270 10.1111/jonm.13383 PMC8237073

[36] Edenlbeld M, Heler O. The corona crisis: characteristics of the unemployed and those returning to the labor market in the first wave and the unemployed in the second wave, National Insurance Institute report. 2022. https://www.btl.gov.il/Publications/more_publications/Documents/mashber-corona.pdf. Accessed Feb 2023.

[37] OECD Employment Outlook 2020 Employment: time spent in aid and unpaid work, by sex. 2022. https://stats.oecd.org/index.aspx?queryid=54757. Accessed Feb 2023.

[38] Krakowczyk JB, Planert J, Skoda EM, Dinse H, Kaup T, Teufel M, Bäuerle A. Pandemic fatigue, psychopathological risk factors, and vaccination attitudes during the COVID-19 pandemic in 2021-a network analysis. J Affect Disord Rep. 2022;8:100345. 10.1016/j.jadr.2022.100345. 35382495 10.1016/j.jadr.2022.100345 PMC8969297

[39] Ali-Saleh O, Obeid S. Compliance with COVID-19 preventive guidelines among minority communities: the case of Israeli Arabs. J Racial Ethn Health Disparities. 2022;9:1–12. 10.1007/s40615-022-01344-0. 35679011 10.1007/s40615-022-01344-0 PMC9179224

[40] Al-Sowayan BS, Mohd Zin NK, AlBdah B, Aldosari SM, Alshareeda AT. Gender differences in response towards COVID 19-related content on social media. Front Womens Health 2021;6. 10.15761/FWH.1000206

